# Bilberries: Curative and Miraculous – A Review on Bioactive Constituents and Clinical Research

**DOI:** 10.3389/fphar.2022.909914

**Published:** 2022-06-29

**Authors:** Zuzana Vaneková, Judith M. Rollinger

**Affiliations:** ^1^ Department of Pharmacognosy and Botany, Faculty of Pharmacy, Comenius University, Bratislava, Slovakia; ^2^ Department of Pharmaceutical Sciences, Division of Pharmacognosy, University of Vienna, Vienna, Austria

**Keywords:** *Vaccinium myrtillus*, bilberry, phenolic compounds, diabetes mellitus, night vision, circulatory disorders

## Abstract

Bilberry (*Vaccinium myrtillus* L.) fruits are an important part of local diets in many countries and are used as a medicinal herb to treat various disorders. Extracts from fruits are often a part of eye health-promoting supplements, whereas extracts from leaves are advertised for type 2 diabetes mellitus and glycemic control. This review provides an overview of the current knowledge of the phytochemical contents of bilberry fruits and leaves and their bioactivities, critically summarizes origins of the health claims and the outcome of clinical trials, with special attention towards those published in the past 10 years. Overall, the three most referenced indications, which are type 2 diabetes mellitus, vision disorders and circulatory diseases, all include contradictory results with no clear conclusion as to the benefits and recommended dosages. Moreover, the indications for vision disorders and diabetes originate from unproven or false claims that have been repeated in research since the 20^th^ century without consistent fact-checking. Beneficial clinical results have been attested for the treatment of dyslipidemia and chronic inflammatory disorders when applied as dietary supplementation of fresh bilberries or as anthocyanin-rich bilberry fruit extracts. However, there is a general lack of double-blinded controlled research with larger sample sizes.

## 1 Introduction

Bilberry (*Vaccinium myrtillus* L.) fruits are an important part of local diets in many countries. They are valued for their pleasant taste and are often processed into jams, preserves, pies, juices and alcoholic beverages. Their high market value is caused by their relatively difficult availability. Bilberry bushes only grow in wild, montane areas. It is not possible to cultivate them due to very specific soil demands. Picking the berries is a tedious and tiring work either using hands or small harvesting combs (Zoratti et al., 2016; Vaneková et al., 2020).

Bilberries have also been used as a medicinal herb to treat various disorders. The extracts from the fruits and leaves have a long-standing tradition of use for vision-related ailments, elevated blood sugar levels and several different cardiovascular disorders. The popularity of bilberries is increasing along with the marketing claims of being “functional food” or “superfood” and various health claims that are not always substantiated. To promote an herbal supplement either not backed by research at all or with only superficial proof might offer no benefits to the consumer or, at worst, cause serious harm to the health. Therefore, it is important to keep track of the recent findings in the field of ethnopharmacology.

Moreover, herbal supplements containing bilberry extracts are not subject to detailed quality control due to the generally lax legislation. Although the quality specifications for bilberries are regulated by three monographs of the European Pharmacopoeia (Bilberry fruit, fresh; Bilberry fruit, dried; Fresh bilberry fruit dry extract, refined and standardized) (European Directorate for the Quality of Medicines & HealthCare 2019) herbal supplements do not have to comply to the pharmacopoeial requirements. Indeed, as recently analyzed by Gaspar et al. (2021), supplements found in pharmacies and health food stores are often adulterated with anthocyanins from other sources, with other cheaper berry extracts, or they do not contain any beneficial extracts at all.

This review summarizes the current knowledge of the chemistry and bioactivities of the constituents of bilberry fruits and leaves. It aims to critically assess the origins and the level of scientific evidence behind the above-mentioned health claims with a special focus on clinical studies performed using bilberry fruits and leaves or extracts thereof.

## 2 Botanical Characteristics of Selected *Vaccinium* Species

### 2.1 *Vaccinium myrtillus L.*

(Syn. *Myrtillus niger* Gilib., *Myrtillus sylvaticus* Drejer, *Vaccinium oreophilum* Rydb., *Vitis-idaea myrtillus* (L.) Moench) (The Plant List, 2022).

Common bilberry is a small bush, 30–50 cm high, densely branched with erect green triangular twigs. Leaves are deciduous, bright green, 1–3 cm long with short petiole, ovate, with serrulate margin. Flowers singular, growing from axillary buds, with short pedicels, blooming in April–June. Sepals and petals five each, connate and forming an urceolate corolla, white with greenish or reddish hues. Stamens 8–10, anthers with two horn-like hollow appendages. The fruit is a round berry with persisting style, 5–10 mm wide, dark blue and glaucous. The pulp is as dark as the peel, the taste is sweet and astringent (Bertová, 1982). A rare albino form has greenish white fruits; the light color is caused by suppression of genes that code the anthocyanin synthesis (Zorenc et al., 2016).

Bilberry is a chamaephytic plant (plant that bears hibernating buds on persistent shoots near the ground), grows on acidic, wet, humous, rocky or bog soils. It spreads through seed dispersal as well as dense system of rhizomes (Bertová, 1982; Kliment and Valachovič 2007). In arctic and subarctic regions it characteristically grows in moist boreal forests dominated by Norway spruce, abundantly from the west coast of Northern Europe to Caucasus toward the northern Asia Pacific coast. In lower latitudes of Europe it can be found in dryish upland forests, heaths, and mountains. A few disjunct populations have been reported in western North America and central Japan (Zoratti et al., 2016).

### 2.2 Blueberries

‘Blueberry’ is an umbrella term which often causes confusion in nomenclature and can include several species of wild and cultivated *Vaccinium* plants, mainly of North American origin. It most commonly refers to *V. corymbosum* L. (northern highbush blueberry), *V. angustifolium* Aiton (common lowbush blueberry), *V. ashei* Reade. (rabbiteye blueberry), their cultivated varieties and various hybrids. The exact taxonomic differentiation is extremely difficult, as almost every single hybrid and variety has been pronounced a standalone species at some point. Out of the above-mentioned, *V. corymbosum* is the one with the highest commercial yields (Kole, 2011).

Northern highbush blueberry is a tall, erect, deciduous bush 0,5–2 m tall, twigs are yellow-green and lenticellar. Leaves alternate, ovate, 3–8 cm long, margin entire to serrulate, color is dark green and changes to red in autumn. Flowers are white or sometimes reddish white, narrowly urceolate, 1 cm long, in terminal racemes. Bloom is in spring, and fruits are round berries, with dark blue and glaucous peel and greenish white pulp, 1–2 cm in diameter and sweet.

This species originates from the eastern United States and Canada where it grows on acidic soils of heathlands, margins of lakes and rivers, swamps, bogs, meadows, and forests. It is cultivated all over the world as a commercial fruit bush, in many different cultivars and hybrids (Horáček 2007; Flora of North America, 2022).

### 2.3 Other Related Species

The second most common species of the *Vaccinium* genus in Europe is the common lingonberry (*Vaccinium vitis-idaea* L.). Lingonberry fruit is a round berry, the peel is red and covered in shiny wax layer; the pulp is white and has a sour and astringent taste (Bertová, 1982; Horáček 2007). Lingonberry is a chamaephytic, often creeping bush, growing on acidic and humous soils in boreal, subalpine and alpine zones (Bertová, 1982; Kliment and Valachovič 2007), used similarly to cranberry (*Vaccinium oxycoccos* or *V. macrocarpon*) because of its similar taste and aroma, albeit more sour and astringent.

*Vaccinium × intermedium* Ruthe is a rare natural hybrid of bilberry and lingonberry. It was experimentally proven that it is an offspring of lingonberry pollen and a flowering bilberry plant. The biggest barrier to hybridization lies in different flowering times–bilberry usually flowers several weeks earlier than lingonberry when they grow in the same location.

Bilberry and lingonberry display some substantial differences in their growth strategies and morphology, which are visible as intermediate characteristics in the hybrid. In the hybrid some leaves drop in the fall and others overwinter. The fruits of the hybrid grow single in branches, similarly to bilberries, whereas lingonberries grow in clusters. The hybrid species produces notably less flowers and smaller fruits than the parent plants and the colour is dark but has more reddish hue (Lätti et al., 2011).

The species most commonly mistaken for bilberry in its natural habitat is bog bilberry (*Vaccinium uliginosum* L.). It is a bog species frequent in low heathlands and montane moorlands of Europe, Asia and North America. The fruit is a dark blue round berry with whitish pulp (Bertová, 1982; Horáček 2007). They are universally considered edible and nutritious, however local sources in Europe mention the folklore names “Rauschbeere” (DE: “intoxicating berry”), “šialenica” (SK: “mad berry”), “blinkavka” (CZ: “vomit berry”) or „opilki” (PL: “drunken berry”) and accompanying effects after consumption: headaches, nausea, vomiting and hallucinations. This is unlikely to be caused by the berry itself, rather possibly by a species of parasitic fungus *Monilinia megalospora* (Woronin) Whetzel., which forms mycelia in ripe and fallen berries, turns them into sclerotia and forms the apothecium the following spring (Woronin 1888; Kotlaba and Pilát 1952; Rätsch 2005). This is, however, merely a hypothesis, as it has not been proven by research. There are currently no studies regarding the potential fungal metabolites or their effects in the scientific literature.

## 3 Bioactive Constituents of *Vaccinium myrtillus* Fruits and Leaves

### 3.1 Phenolic Compounds

#### 3.1.1 Anthocyanins

**FIGURE 1:**
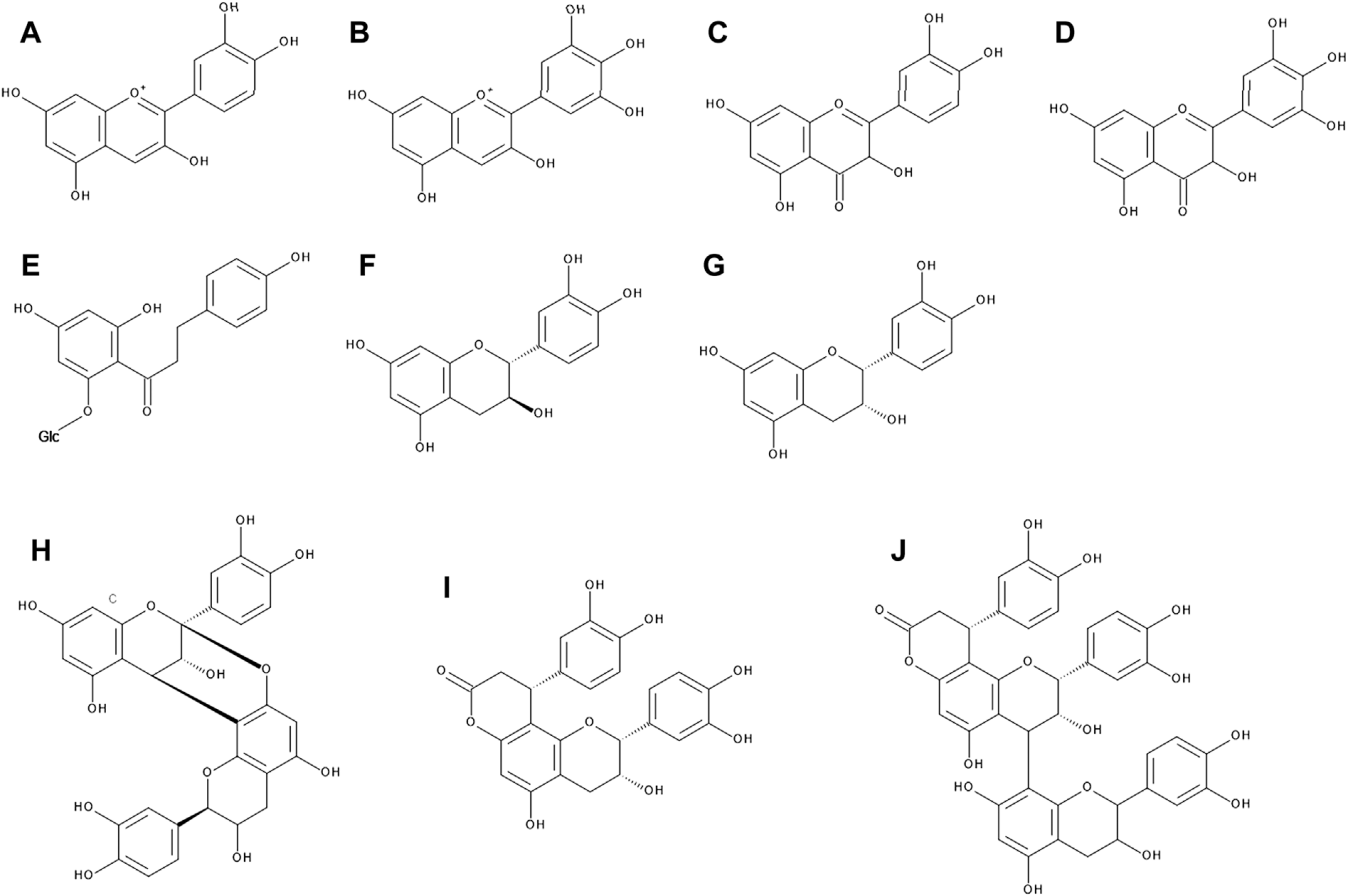
The chemical structures of cyanidin **(A)**, delphinidin **(B)**, quercetin **(C)**, myricetin **(D)**, phlorizin **(E)**, catechin **(F)**, epicatechin **(G)**, A-type procyanidin dimer **(H)**, cinchonain Ia **(I)**, and cinchonain IIa **(J)**.

The fruits of each above listed species have a characteristic anthocyanin profile which can be used for fingerprint analysis:

• *V. myrtillus* contains mainly delphinidin and cyanidin (Figure 1) in a 1 : 1 ratio, followed by petunidin, peonidin and malvidin
• *V. vitis-idaea* contains mainly cyanidin (>90%) and small amounts of peonidin, other anthocyanins are absent
• *V. × intermedium* typically contains more cyanidin compared to bilberry
• *V. corymbosum* contains mainly delphinidin, peonidin is absent
• *V. uliginosum* contains delphinidin and malvidin in 1 : 1 ratio (Määttä-Riihinen et al., 2004)

*V. myrtillus* has the most intensely colored berries out of all above mentioned species. This is due to the fact that both peel and pulp contain large amount of anthocyanidins (up to 2% of the fresh weight in peels), whereas in other species the pulp is white or light pink at most (Riihinen et al., 2008). In bilberries, anthocyanins comprise about 90% of the total phenolic compounds of the fruit (Kähkönen et al., 2003; Heinonen 2007). In nature, anthocyanins occur mainly in glycosylated form. The most common types in fruits of *Vaccinium* genus are glucosides, galactosides and arabinosides (Määttä-Riihinen et al., 2004; Lätti et al., 2011; Colak et al., 2016). Fruits of *V. myrtillus* contain unique cyanidin- and delphinidin-3-*O*-sambubiosides where the saccharide moiety is glucose (2→1) xylose (Du et al., 2004). It is also possible to isolate 3-*O*-methyl anthocyanidins in large amounts from bilberry fruits (Ichiyanagi et al., 2020).

#### 3.1.2 Flavonols

Quercetin is the main flavonol of bilberry fruits, accounting for more than 50% of total flavonoid content (Riihinen et al., 2008; Cardeñosa et al., 2016; Zorenc et al., 2016). The second most abundant one is myricetin (Figure 1); other flavonols, such as syringetin, laricitrin and isorhamnetin, have only been detected in low levels (Mikulic-Petkovsek et al., 2014; Zorenc et al., 2016). Kaempferol, although abundant in bilberry leaves (Riihinen et al., 2008; Hokkanen et al., 2009; Cardeñosa et al., 2016), is present in fruits only in trace amounts (Zorenc et al., 2016). Flavonols in *V. myrtillus* occur mainly in glycosylated form. Studies determined various hexosides, pentosides and glucuronides. The most abundant glycosides appear to be rhamnosides and glucuronides (Zorenc et al., 2016). Phlorizin, a glucoside of a dihydrochalcone phloretin, was found in bilberry fruits, however the aglycone itself was not detected (Ancillotti et al., 2016).

#### 3.1.3 Tannins and Flavanols

Plants of the entire *Vaccinium* genus are one of the richest sources of tannins, of both hydrolyzable and condensed type, as well as their various derivatives. Catechin and epicatechin have been found in both leaves and fruits in abundance (Hokkanen et al., 2009; Može et al., 2011; Ancillotti et al., 2016; Zorenc et al., 2016), a B-type dimer of catechin was found in fruits (Määttä-Riihinen et al., 2005). Gallocatechin and epigallocatechin can be found predominantly in leaves (Hokkanen et al., 2009; Zorenc et al., 2016). Both leaves and fruits of *V. myrtillus* are especially rich in procyanidin, its various dimers and trimers. The universal B-type procyanidins have a single bond between structural units of catechins, whereas the rare A-type procyanidins are double bonded. The A-type linkages (Figure 1) dominate in bilberries over the B-type, which is uncommon in other plant species (Määttä-Riihinen et al., 2005; Riihinen et al., 2008; Hokkanen et al., 2009; Ancillotti et al., 2016).

#### 3.1.4 Cinchonains

Various isomers of the flavonolignans cinchonains I and II (Figure 1) were detected in leaves and stems of *V. myrtillus* (Hokkanen et al., 2009; Bujor et al., 2016). However, in the fruits only cinchonain I isomers were found (Bujor et al., 2016).

#### 3.1.5 Coumarins

The existence of the coumarins, namely umbelliferone (Tumbas Šaponjac et al., 2015) and esculetin (Ancillotti et al., 2016), have been reported from bilberry fruits.

#### 3.1.6 Arbutin and Hydroquinone

**FIGURE 2:**
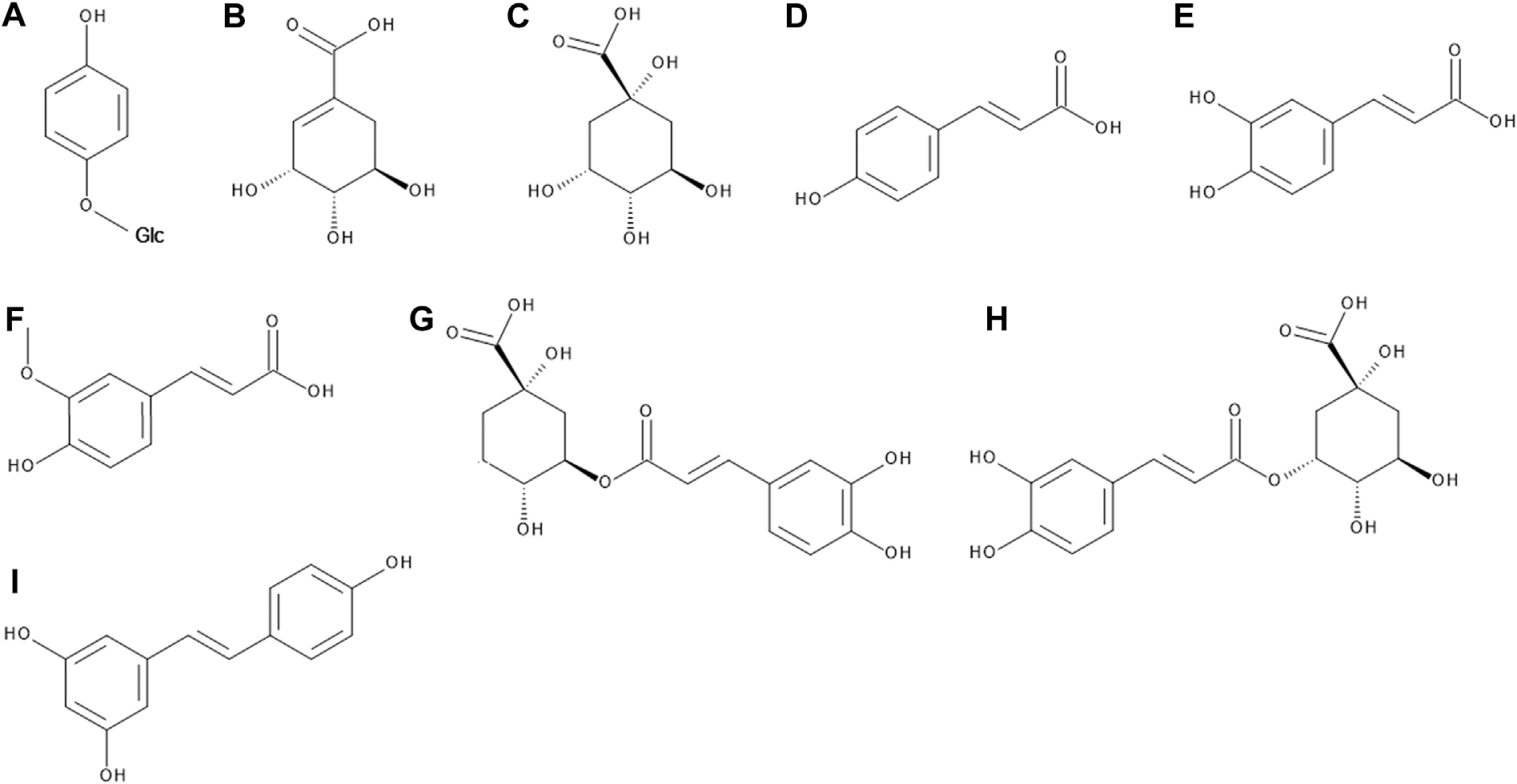
The chemical structures of arbutin **(A)**, shikimic acid **(B)**, quinic acid **(C)**, *p*-coumaric acid **(D)**, caffeic acid **(E)**, ferulic acid **(F)**, chlorogenic acid **(G)**, neochlorogenic acid **(H)**, and resveratrol **(I)**.

The presence of arbutin (Figure 2) in the leaves *V. myrtillus* L. has always been widely debated. Ramstad explored the claims in several printed sources from the first half of the 20^th^ century that the drug contains arbutin and concluded that these claims do not seem to be based upon chemical investigation of the plant but rather inferred by virtue of a close botanical relationship of *V. myrtillus* to other arbutin-bearing species such as *Arctostaphylos uva-ursi* and *V. vitis-idaea* (Ramstad 1954). Also Von Friedrich and Schönert did neither prove the presence of arbutin nor hydroquinone in *V. myrtillus* (Von Friedrich and Schönert 1973). Sticher et al. used HPLC to prove the absence of arbutin, hydroquinone and their derivatives in *V. myrtillus* (Sticher et al., 1979). Rychlinska and Nowak optimized the HPLC method for proving arbutin and hydroquinone simultaneously and identified them in the leaves of *Arctostaphylos uva-ursi* and *V. vitis-idaea*, but not in leaves of *V. myrtillus* and *V. uliginosum* (Rychlinska and Nowak 2012). Two separate research teams came to the same conclusion also using HPLC (Ieri et al., 2013; Ștefănescu et al., 2020). Hokkanen et al. identified arbutin in leaves of lingonberry and its hybrid *Vaccinium × intermedium*, but not in bilberry (Hokkanen et al., 2009).

Contrarily, two studies describe the determination of arbutin in bilberry leaves using spectrophotometry (Rosłon et al., 2011) or HPLC (Stefkov et al., 2014) but those might suffer from mistakes in their methodology. Overall, studies agree that *V. myrtillus* does not contain any arbutin unless hybridized with lingonberry.

#### 3.1.7 Phenolic, Hydroxycinnamic and Other Organic Acids

Bilberry leaves and fruits are an abundant source of organic acids and their derivatives. Their content and exact structures have been researched in great detail in the last 15 years. The studies generally agree that they comprise the majority of the total phenolic compounds in the leaves, whereas in the fruits they are overshadowed by anthocyanins (Riihinen et al., 2008; Bujor et al., 2016). The simple organic acids found in bilberry fruits include shikimic, quinic, citric and malic acid (Mikulic-Petkovsek et al., 2014). As for phenolic and hydroxycinnamic acids, their composition is dominated by *p*-coumaric, caffeic and ferulic acid (Hokkanen et al., 2009; Tumbas Šaponjac et al., 2015; Ancillotti et al., 2016; Zorenc et al., 2016), followed by syringic acid (Tumbas Šaponjac et al., 2015), gallic and ellagic acid (Može et al., 2011; Ancillotti et al., 2016). In a minority, vanillic acid (Juadjur et al., 2015), dihydroxybenzoic acid (Borowiec et al., 2014) and salicylic acid (Ancillotti et al., 2016) can be found in bilberry fruits.

Above listed organic acids can be detected in these plants either free, esterified, or etherified. They form esters either with each other or with other phenolic compounds. Etherification usually occurs with various saccharide moieties (Ieri et al., 2013; Bujor et al., 2016). The most notable out of these derivatives are chlorogenic and neochlorogenic acid (Figure 2), which often make up the majority of the total phenolic acid content of bilberry fruits and leaves (Riihinen et al., 2008; Ancillotti et al., 2016; Bujor et al., 2016).

#### 3.1.8 Stilbenoids

Resveratrol (Figure 2) was found in the bilberry fruits by two different research groups (Rimando et al., 2004; Može et al., 2011) and in the leaves (Tadić et al., 2021). It is worth noting that while cultivated blueberries contain both resveratrol and piceatannol, the latter is missing in bilberries completely (Rimando et al., 2004). The presence of pterostilbene is still being debated; some report a complete lack of it (Rimando et al., 2004), others report its presence in bilberry fruit extracts (Habanova et al., 2016; Waszczuk et al., 2020).

### 3.2 Terpenoids

#### 3.2.1 Triterpenoids

**FIGURE 3:**
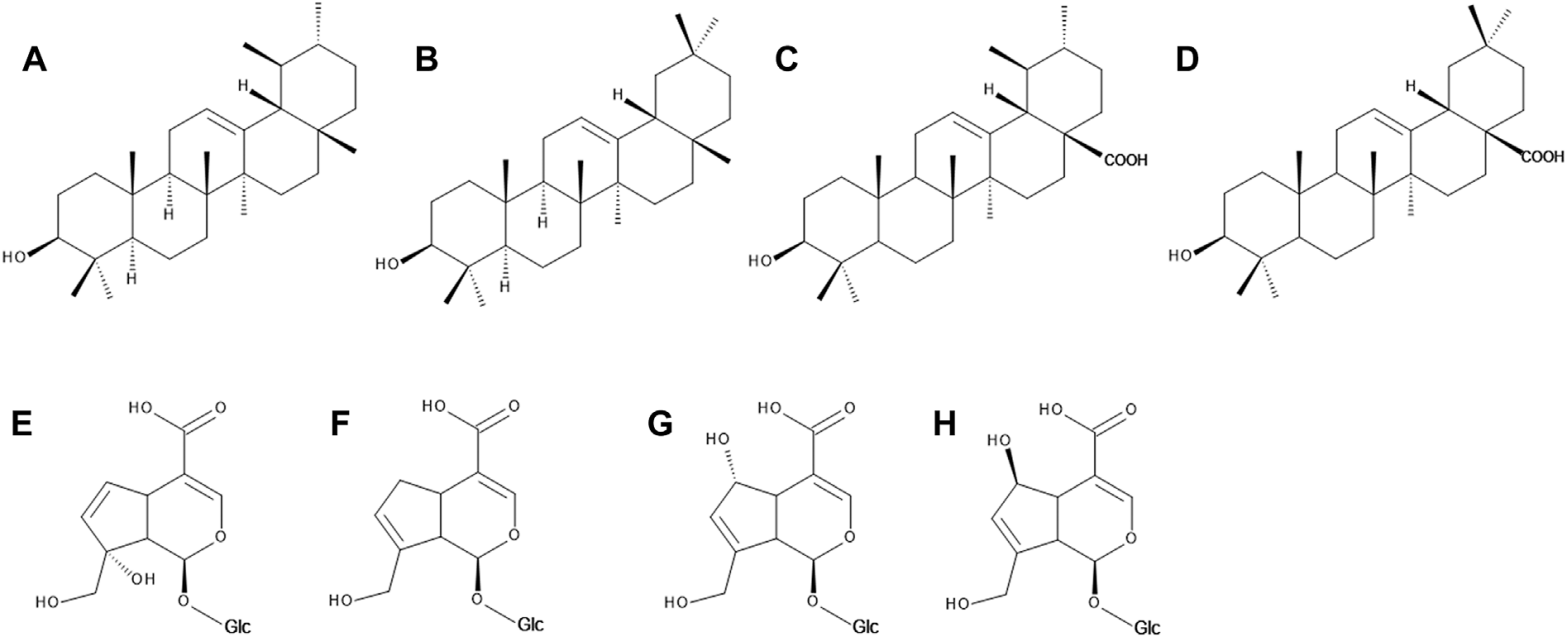
The chemical structures of α-amyrin **(A)**, ß-amyrin **(B)**, usolic acid **(C)**, oleanolic acid **(D)**, monotropein **(E)**, geniposidic acid **(F)**, deacetylasperulosidic acid **(G)** and scandoside **(H)**.

The whitish wax cuticle covering the bilberry fruits consists mainly of triterpenoids such as α- and ß-amyrin, oleanolic and ursolic acid (Figure 3), as well as fatty cerotic acid, sitosterol and 2-heneicosanone. The wax layer of bilberries is similar to that of blueberries and much thinner than that of lingonberries and crowberries (Dashbaldan et al., 2019; Trivedi et al., 2019). Oleanolic and ursolic acid, α- and ß-amyrin, lupeol, lupenyl acetate and betulin were identified in the extract of bilberry leaves (Vrancheva et al., 2021).

#### 3.2.2 Tetraterpenes

ß-Carotene, as well as the xanthophylls lutein, zeaxanthin and ß-cryptoxanthin were found in bilberry fruits (Mahdavi et al., 1998; Bunea et al., 2012). The bilberry seeds, as well as the oil exctracted from them, is rich in vitamin E, notably γ-tocotrienol (Gustinelli et al., 2018).

#### 3.2.3 Iridoids

In *V. myrtillus* monotropein, dihydromonotropein and deacetylasperulosidic acid (Figure 3) were identified either as standalone compounds (Jensen et al., 2002; Heffels et al., 2017), or as derivatives, such as *p*-coumaroyl monotropein, p-coumaroyl dihydromonotropein (Bujor et al., 2016), *p*-coumaroyl-deacetylasperulosidic acid (Heffels et al., 2017). 10-*p*-Trans-coumaroyl-1S-monotropein is an iridoid glycoside found in bilberry fruits which subsequently got a name vaccinoside (Aaby et al., 2013). Scandoside, its *p*-coumaroyl derivative (Heffels et al., 2017) and geniposidic acid (Borowiec et al., 2014) were reported in the bilberry fruits. Several studies also report on a significant number of various iridoid derivatives which, however, could not be further identified (Hokkanen et al., 2009; Ieri et al., 2013; Juadjur et al., 2015; Zorenc et al., 2016; Heffels et al., 2017).

### 3.3 Other Constituents

Xyloglucans are heteropolysaccharides with mechanical function. In plants they form bridges between cellulose microfibrils in the cell wall. In bilberry fruits they consist of xylose, glucose, galactose and fucose (Hilz et al., 2007).

**FIGURE 4:**
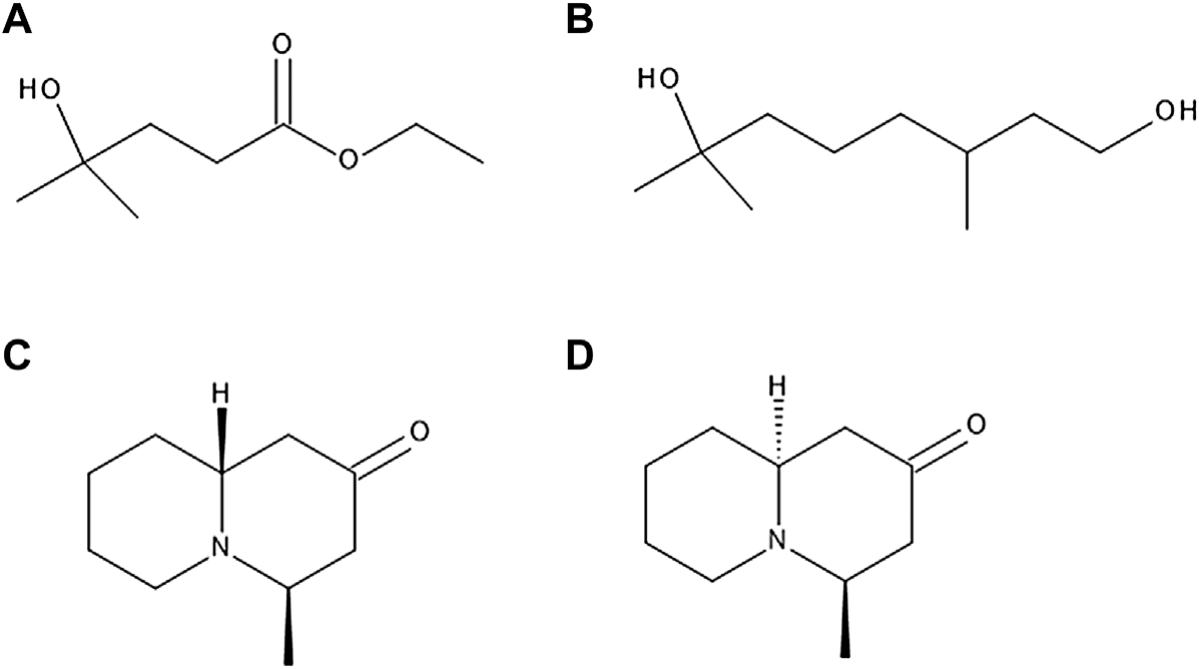
The chemical structures of ethyl 3-hydroxy-3-methylbutanoate **(A)**, hydroxycitronellol **(B)**, myrtine **(C)** and epimyrtine **(D)**.

A study regarding volatile compounds of *V. myrtillus*, *V. corymbosum* and others determined that out of a vast number of volatile compounds present in these fruits, the ones with most prominent blueberry-like aroma were ethyl 3-hydroxy-3-methylbutanoate, a few other esters with similar structure, and hydroxycitronellol (Figure 4). Other compounds responsible for the aroma were identified as *trans*-2-hexenal, *trans*-2-hexenoic acid, phenylacetaldehyde, methyl salicylate and 2-phenylethyl formate (Hirvi and Honkanen 1983).

Alkaloids myrtine and epimyrtine (Figure 4) were detected in above-ground parts of bilberry plants (Slosse and Hootelé 1981). Another, more recent study confirmed these findings (Nardin et al., 2017).

## 4 Uses of *Vaccinium myrtillus* L.

### 4.1 Dietary, Technical and Industrial Uses

Wild berries are a valuable part of the European nature and tradition. Especially in the northern and eastern parts of Europe, wild berries grow abundantly, and berry picking is an important form of recreation for many people. In these areas, about half of the wild edible berries are picked for personal consumption and the other half for commercial use. They are picked either by hand or by traditional wooden or metal combs. The average bilberry yield in Nordic countries has been estimated to account for more than 500 million kg per year, of which only 5–8% is currently exploited. The wild berry industry in Europe is typically fairly small and fragmented. The distinctions in the forest policies, access rights, and the berry picking traditions also differ to some extent between European countries.

To secure the availability of bilberries for commercial exploitation, methods for field cultivation and semi-cultivation of natural stands have been considered. Semi-cultivation of wild stands of *Vaccinium* spp. has been a great success in North America for decades. The North American lowbush blueberry, *Vaccinium angustifolium* Aiton, is semi-cultivated using modern agricultural practices such as fertilization, pruning, and mechanical harvesting. Attempts to cultivate and semi-cultivate the bilberry have been initiated in Denmark, Norway, and Finland, with modest results, because of the plant’s specific environmental demands (Nestby et al., 2011; Zoratti et al., 2016).

Most bilberries picked commercially in Northern Europe are exported frozen and unprocessed to East Asia or to the local food industry. China and Japan have been the major customers, increasingly focusing on health products. In their home countries and abroad alike, bilberries are processed into juices, jams, preserves and other highly demanded products (Kresánek and Kresánek 2008; Zoratti et al., 2016). Other industrial uses include the extraction of anthocyanins as food colorants, utilizing the press cake after juicing and extraction of oil from bilberry seeds (Pires et al., 2020).

### 4.2 Pharmacological Activities of *V. myrtillus* Fruit Extracts

The bilberry fruits and fruit extracts are currently on the market as herbal medicinal products with traditional use, according to two herbal monographs by European Medicines Agency (EMA): Bilberry fruit, fresh, which also includes its standardized dry extract (European Medicines Agency, 2015b), and Bilberry fruit, dried (European Medicines Agency 2015c); whereas the former is a herbal drug to relieve symptoms of discomfort and heaviness of legs related to minor venous circulatory disturbances and to relieve symptoms of cutaneous capillary fragility, the latter is applied for symptomatic treatment of mild diarrhoea and minor inflammations of the oral mucosa. The analytic quality of these herbal drugs is regulated by their respective monographs in the European Pharmacopoeia, with an additional monograph for Fresh bilberry fruit dry extract, refined and standardized (European Directorate for the Quality of Medicines & HealthCare 2019).

Natural Standard Research collaboration assessed the clinical evidence on bilberry and its extracts for several indications including diabetes mellitus, glaucoma, retinopathy, cataracts and night vision in 2009. All indications have been assessed with grade C—„Unclear or conflicting scientific evidence”, except for night vision, which was assessed one grade lower, D—„Fair negative scientific evidence“ (Ulbricht et al., 2009).

As there has been an abundance of new clinical studies in the recent years (Supplementary Table S1), these indications will be critically reviewed below.

#### 4.2.1 Antioxidant Activity

Berries of all *Vaccinium* species contain unusually high levels of antioxidant compounds, which effectively makes them valuable food for dietary supplementation of antioxidant compounds. *V. myrtillus* is mainly characterised by delphinidin and cyanidin glycosides together with quercetin and chlorogenic acid (Ancillotti et al., 2016). When studying the antioxidant activities of the whole extract, a strong synergistic effect was apparent, as most of the measured effects were associated not with the anthocyanin fractions, but with the unfractioned extract (Juadjur et al., 2015).

Recent studies have suggested that anthocyanins and other polyphenols can also have indirect effects by stimulating antioxidative defence mechanisms via induction of enzymes such as GST or GSH-Px (Juadjur et al., 2015) or DAF-16 and HSF-1 (González-Paramás et al., 2020). These mechanisms are usually dose dependent. However, a study on isolated rat hearts showed that high concentrations of bilberry anthocyanins (5–50 mg/L) can have diminished cardioprotection and show cardiotoxic activity despite having their radical scavenging and intracellular antioxidant capabilities increased in a concentration-dependent manner (Žiberna et al., 2010).

Antioxidant activity is prerequisite to many beneficial effects in human body: bilberry extracts inhibit oxidative modification of human LDL *in vitro*, inhibit lipid peroxidation in rat liver microsomes and liver lipid peroxidation *in vivo* in mice (Tumbas Šaponjac et al., 2015), attenuate liver damage induced by CCl4 or stress in rats (Bao et al., 2010; Domitrović and Jakovac 2011; Popović et al., 2016), ameliorate oxidative stress after ischaemia-reperfusion injury in isolated rat heart (Žiberna et al., 2010), during metabolic syndrome (Shi et al., 2017) or in cancer cells (Tumbas Šaponjac et al., 2015).

#### 4.2.2 Anti-inflammatory Activity

Drawing from its powerful content of antioxidants, bilberry fruits and their extracts have been investigated for their anti-inflammatory activities as well. Bilberry extract Mirtoselect, standardized to contain 40% of anthocyanins, has been found to induce a complex anti-inflammatory response in lipopolysaccharide-activated macrophages. The response consists of attenuation of pro-inflammatory cytokines (including TNF- α, IL-1β, IL-6, and COX-2), attenuation of multiple lipopolysaccharide-induced chemokines and IL receptors almost to control levels (Chen et al., 2008). In human THP-1 monocytic cells, the response of the cells treated with anthocyanin-rich bilberry extract to inflammatory stimulation was more varied—bilberry extract attenuated most of the IFN-γ-induced signal protein activation, pro-inflammatory gene expression, and cytokine secretion, whereas it enhanced TNF-α-induced responses (Roth et al., 2014). This suggests a distinct role of anthocyanins in the inflammatory modulation that might vary from target to target.

These *in vitro* results are reflected in several clinical studies for inflammatory diseases. For example, after only 7 days of consuming 500 g bilberries per day, the patients suffering from gingivitis showed reduced inflammatory cytokine levels and less bleeding on probing, which is a routine dental clinical parameter of inflammation (Widén et al., 2015). In a study on patients with increased cardiovascular risk, after 4 weeks of consuming 330 ml of bilberry juice daily, some of the participants‘ inflammatory blood markers significantly decreased compared to placebo group (C-reactive protein, IL-6, IL-15, and monokine induced by INF-γ), while other remained unchanged. Unexpectedly, an increase of TNF-α was also observed (Karlsen et al., 2010).

In test subjects with metabolic syndrome, supplementing 400 g fresh bilberries daily for 8 weeks into their diet caused reductions in several inflammatory parameters (C-reactive protein, IL-6, IL-12, and LPS) and generally lower inflammation scores (Kolehmainen et al., 2012). Similar anti-inflammatory results were achieved in metabolic syndrome patients by a supplement containing purified bilberry and blackcurrant anthocyanins, therefore it can be hypothesized that they are the main active principle behind the favorable effects of bilberries on cardiometabolic risk factors (Aboonabi et al., 2020).

Inflammatory bowel diseases are not always satisfactorily treated with standard therapy regimens and the current evidence suggests that a dysregulated immune response to intestinal microbiota induces the relapsing inflammation. *In vitro* experiments, bilberry fruit extract and the isolated anthocyanins were able to attenuate the inflammatory response of human colon epithelial cell cultures stimulated by IFN-γ/IL-1β/TNF-α (Triebel et al., 2012). In patients with mild to moderate ulcerative colitis, an open, non-blinded and non-controlled trial showed that 90,9% subjects showed a positive response to a preparation made of dried bilberry powder and concentrated bilberry juice, with 63,4% patients achieving remission (Biedermann et al., 2013). This makes bilberries a promising therapeutic for inflammatory intestinal disorders and further studies on mechanisms, as well as randomized blinded trials are warranted.

However, the anti-inflammatory effect of bilberries might not be universally applicable. It was observed that the consumption of bilberry juice before, during and 2 days after a half marathon race paradoxically caused a mild increase of exercise-induced muscle soreness and of C-reactive protein levels in blood (Lynn et al., 2018).

#### 4.2.3 Use for Type 2 Diabetes Mellitus

Type 2 diabetes mellitus (T2DM) is a complex disease characterised by hyperglycaemia with an antecedent phase of insulin resistance, often associated with dyslipidemia, increase in pro-inflammatory cytokines and oxidative stress.

According to current studies, the possible pathways of bilberries’ *in vitro* antidiabetic activity are most likely complex and working in synergy. It has been suggested that tannins may have therapeutic potential in the treatment of T2DM, mainly through two ways: they may lower glucose levels by delaying intestinal glucose absorption through inhibition of intestinal α-glucosidase, and they may delay the onset of T2DM by regulating the antioxidant environment of pancreatic β-cells (Shi et al., 2017). Myricetin is a flavonoid abundant in berries and it was reported that the anti-diabetic effectiveness of myricetin is due to its anti-inflammatory activity (Wu et al., 2016). Anthocyanins have been demonstrated to significantly reduce glucose production by 24–74% in H4IIE hepatocytes (Shi et al., 2017). Abscisic acid is another promising compound with antidiabetic properties. It is a plant signalling molecule that plays an important role in fruit ripening and seed development. It has been shown to up-regulate the peroxisome proliferator-activated receptor, PPAR γ, both *in vitro* and *in vivo*. Abscisic acid has been shown to ameliorate the symptoms of T2DM after oral administration, targeting PPAR γ in a similar manner than the thiazolidinediones class of anti-diabetic drugs. It is present in most plant tissues (Bassaganya-Riera et al., 2010). Bilberry fruits contain the highest level of abscisic acid just before the fully ripe stage (38 μg/g dry weight) but fully ripe fruits still contain a considerable amount of this metabolite (13 μg/g dry weight) which can add to the overall antidiabetic effect (Karppinen et al., 2013). Cinchonain Ib has been shown to increase plasma insulin levels in a way similar to glibenclamide *in vitro* and *in vivo* after oral administration (Qa'Dan et al., 2009). It has been theorized that the various cinchonains found in leaves of *V. myrtillus* could be partially responsible for the blood glucose lowering effects (Hokkanen et al., 2009).

In a murine model, the anthocyanin-rich bilberry fruit extract significantly reduced the blood glucose concentration and enhanced insulin sensitivity. AMPK was activated in white adipose tissue, skeletal muscle, and the liver of diabetic mice. This activation was accompanied by upregulation of glucose transporter 4 in white adipose tissue and skeletal muscle and suppression of glucose production and lipid content in the liver. At the same time, acetyl-CoA carboxylase was inactivated and PPARα, acyl-CoA oxidase, and carnitine palmitoyltransferase-1A were upregulated in the liver (Takikawa et al., 2010). Similarly, three different bilberry fruit extracts reduced the trehalose level in the hemolymph of sugar-fed *Drosophila melanogaster* to 50% compared to the control group (Neamtu et al., 2020).

Generally speaking, a high dietary intake of anthocyanins and flavonoids has been associated with a decreased insulin resistance in women of all ages by a large cohort study (Jennings et al., 2013). In line with this result, the dietary anthocyanin consumption has been associated with a 15% reduction of T2DM risk in a large meta-analysis (Guo et al., 2016).

In clinical trials using *V. myrtillus* specifically, oral administration of bilberry fruits and extracts thereof yielded mixed results. Saccharide-free extracts tended to show better antidiabetic effect than whole fruits or fruit juices, most likely due to the higher content of biologically active substances instead of sugars, which are present in berries in considerable quantities (Shi et al., 2017). One study showed that patients with T2DM who ingested a single oral dose of bilberry fruit extract (standardized 36% anthocyanins) had lower postprandial insulin and glucose blood levels than the placebo group (Hoggard er al. 2013). Another study, where patients added 400 g fresh bilberries to their diet for 8 weeks, showed an inverse correlation between dietary intake of bilberries and fasting plasma glucose level, but insulin sensitivity remained unchanged (De Mello et al., 2011).

However, other clinical studies have indicated that there were no significant differences in fasting plasma glucose between the treatment and the control groups after dietary supplementation with anthocyanins for 12 (Qin et al., 2009) or 24 weeks (Zhu et al., 2013), or after ingesting 400 g fresh bilberries daily for 2 months (Kolehmainen et al., 2012). Even the newest clinical study on Chinese patients with T2DM using 1400 mg of bilberry extract per day did not report any significant differences compared to placebo in any of the measured biomarkers (Chan et al., 2021).

#### 4.2.4 Use for Eye-Related Ailments

##### 4.2.4.1 Night Vision

The first record of bilberry fruits for eye issues goes back to World War II when a bilberry jam was allegedly used by British pilots to increase night vision. There exist multiple (mostly unsourced) claims that this was a hoax purposefully created by British to cover up the technological advancement of airplane radar systems (Canter 2004) or purely a product of R.A.F. pilots’ famous superstitions (Bilberries and Night Vision: Reality Check, 2022). However, in the search for a proof, the claims have been since studied in various models.

The fruits were shown to contain lutein, which is commonly used as a supplement to improve eye health (Bunea et al., 2012). However, the anthocyanins are often commonly referred to as the compound class which improves night vision. The outcomes of clinical trials are inconsistent, as summarized by a systematic review of the available clinical studies (Canter and Ernst 2004). The ones using relatively low doses of standardized anthocyanins (12–150 mg daily) did not prove any beneficial effects (e.g., Mayser and Wilhelm 2001). Studies with higher doses (up to 720 mg/day) showed beneficial effects in some measured aspects of night vision, but never enough to conclude the study as successful. One study in particular used 40 military pilots as test subjects with 400 mg bilberry anthocyanins taken before flight. Subjects reported reduced and shorter-lived post-dazzling after-images and reduced subjective visual fatigue but there was no difference in objective ophthalmological values (Belleoud et al., 1966). Overall, the systematic review of the available clinical studies published in 2004 shows that the extract does not improve night vision in healthy eyes, but there is a serious lack of rigorous clinical trials in patients with impaired night vision or diagnosed eye disease. There are plausible mechanisms by which constituents of *V. myrtillus* could modify vision and the health of the eye, including accelerated re-synthesis of rhodopsin, antioxidant activity, modulation of retinal enzyme activity, stabilization of collagen, anti-inflammatory action, and improved microcirculation. Whether the extract has any unique properties over and above those of plant-derived antioxidants remains to be elucidated. There is a need of more studies with subjects suffering impaired night vision due to pathological eye conditions, particularly macular degeneration (Canter and Ernst 2004).

##### 4.2.4.2 Use for Retinopathy

The first study on this topic was an open, placebo-controlled study on patients with diabetic retinopathy; the treatment with 200 mg of unspecified bilberry fruit extract combined with 10 mg beta-carotene taken 3 times daily reduced vascular permeability and improved the state of retinal blood vessels (Scharrer and Ober 1981). It was followed by another study on patients with diabetic and hypertensive retinopathy who took 160 mg of a bilberry extract containing 25% anthocyanidins twice daily. The authors reported on 77–90% improvement (compared to placebo) in ophthalmoscopic and fluoro-angiographic anomalies, but the study, similarly to the previous one, suffers from serious methodological issues (Perossini et al., 1987).

Another study tested the effect of an unspecified bilberry fruit extract given in a dose of 510 mg daily over 1 year on patients with diabetic retinopathy. It reported a gradual improvement in contrast sensitivity, but other measured parameters (corrected visual acuity, hard exudates, microaneurysms, leaking points) remained unchanged for the entire duration of the study (Kim et al., 2008).

##### 4.2.4.3 Use for Other Eye-Related Ailments

A combination of two phenolic extracts from bilberry (standardized to 36% anthocyanins) and French maritime pine bark (standardized to 70% procyanidins) lowered elevated intraocular pressure in patients almost as effectively as latanoprost, however, it took much longer (24 *vs*. 4 weeks). The combination treatment with both extracts and latanoprost was even more effective for lowering intraocular pressure and the combination yielded better retinal blood flow. No serious side effects occurred during the study, apart from common side effects in some of the latanoprost patients (temporarily blurred vision and eyelid redness). However, limitations of this study included a lack of a placebo group, double-blinding and randomization. The use of a multi-ingredient product also makes it difficult to discern the effects of bilberry alone (Steigerwalt et al., 2010).

Twenty-two patients with dry eye symptoms were treated with 160 mg of (unspecified) bilberry nutraceutical. After 30 days, the Ocular Surface Disease Index was statistically improved in the bilberry group compared to placebo (Anderson et al., 2011).

In a double-blind, randomized, placebo-controlled trial, a supplementation of 240 mg standardized bilberry extract daily for 12 weeks significantly improved the tonic accommodation of the ciliary muscle during near-vision tasks on display terminals, and therefore alleviated the ocular fatigue symptoms (Kosehira et al., 2020).

Thirty healthy individuals with myopic eyes have undergone treatment with either 400 mg of yeast-fermented bilberry fruit extract or placebo. After 4 weeks of treatment, there was an improvement in accommodation and mesopic contrast sensitivity, but not in other measured parameters (Kamiya et al., 2013).

#### 4.2.5 Use for Circulatory Disorders

It is well-documented that flavonoids, flavonoid chalcones and other related phenolic compounds have beneficial effects on circulatory disorders (Ulloa 2019). The use of bilberry fruits and anthocyanin-rich extracts for microcirculation issues and peripheral venous insufficiency is supported by the review of European Medicines Agency (European Medicines Agency 2015a). Older studies reviewed in the EMA assessment report show positive results, such as reduction of the number of petechiae on the skin of 27 patients with capillary fragility who took 80–120 mg of bilberry anthocyanosides (Coget and Merlen 1968) or reduction of venous insufficiency symptoms such as oedema, cramp-like pain and paresthesia by a regime of 480 mg bilberry fruit extract daily (Gatta 1988), but are generally lacking in study design and statistical analysis. There is also a lack of newer clinical research on this topic.

#### 4.2.6 Use for Dyslipidemia

Several studies have been conducted addressing the influence on plasma lipoproteins. In humans, a study performed on patients after myocardial infarct showed that bilberry powder (40 g per day) significantly potentiated the effect of statins on plasma total cholesterol and LDL cholesterol. The bilberry group patients were also more successful in a 6 min walking test (Arevström et al., 2019) The same group of patients was also evaluated for the presence of circulating microvesicles which are a marker of cardiovascular disease. It was found that bilberry extract improved the blood profile and reduced endothelial vesiculation (Bryl-Górecka et al., 2020). Another clinical study showed that consumption of 150 g of frozen bilberries 3 times a week for 6 weeks led to a significant decrease in total cholesterol, LDL and triglycerides, and a favorable increase in HDL (Habanova et al., 2016). This is in agreement with an older studies which showed significant decrease in LDL and increase in HDL after supplementing the dyslipidemic patients with 320 mg of purified anthocyanins daily for 12 weeks (Qin et al., 2009) or 24 weeks (Zhu et al., 2013).

#### 4.2.7 Antiproliferative Activity and Anti-Cancer Treatment

Drawing from the numerous studies on antioxidant activity of bilberry extracts, there have been several studies on cancer cell lines. Fractions containing catechins, flavonoids and organic acids were able to inhibit the growth of cervix epitheloid carcinoma, breast adenocarcinoma and colon adenocarcinoma cell lines, with IC_50_ ranging from 125.80 to 300.48 mg/ml (Tumbas Šaponjac et al., 2015). Whole bilberry extract inhibited the growth of three colon cancer cell lines (Caco-2, HT-29, and HCT 116) (Aaby et al., 2013), whole bilberry extract and the standardized mixture of its main phenolic compounds inhibited the growth of prostate cancer cell lines (Del Bubba et al., 2020). The bilberry fruit extract also inhibited early preneoplastic liver cell lesions in rats (Hara et al., 2014). Whole bilberry powder inhibited the viability, proliferation, migration and invasion of oral squamous carcinoma cell lines in zebrafish model (Mauramo et al., 2021).

Only one clinical pilot human study has been published to date. Three different doses of a standardized anthocyanin-rich bilberry fruit extract (1,400, ,2800 or 5,600 mg daily) were given to 25 colorectal cancer patients scheduled for resection for 7 days before the surgery. In all patients, the tumor cell proliferation was decreased by 7% compared to the baseline; the apoptotic index increased from 3.6 to 5.3%, regardless of the dosage. The study however lacks a control group (Thomasset et al., 2009).

#### 4.2.8 Neuroprotective Activity

Cholinesterases are key enzymes participating in the pathogenesis of Alzheimer’s disease and screening for cholinesterase inhibitors in selected fruits and vegetables is a potential way of finding new treatment options. In bilberry fruit extract, the derivatives of chlorogenic and benzoic acid showed the highest cholinesterase inhibition (Borowiec et al., 2014). Bilberry-supplemented rats achieved better results at maze tests and their hippocampal parvalbumin-immunoreactive neurons were significantly reduced (Borowiec et al., 2019). Likewise, supplementing bilberry anthocyanins improved learning and memory abilities of Alzheimer’s disease model mice (Li et al., 2020). Unfortunately, the relevant clinical study, in which the elderly volunteers showed fewer cognitive symptoms and improved memory discrimination, used commercially available North American blueberries, not European bilberries (McNamara et al., 2018).

#### 4.2.9 Effects on Skin Health

A water-soluble *V. myrtillus* fruit extract (total polyphenols 339.3 mg/100 g FW; total anthocyanins 297.4 mg/100 g FW) was able to reduce UVA- and UVB-induced damage in a human keratinocyte cell line. The extract was able to reduce the UVB-induced cytotoxicity and genotoxicity and UVB-induced lipid peroxidation. With UVA-induced damage, *V. myrtillus* reduced genotoxicity as well as the imbalance of redox intracellular status. Moreover, the extract reduced the UVA-induced apoptosis, but had no effect against the UVB one (Calò and Marabini 2014). A cream which incorporated both the oil from bilberry seeds and the extract from bilberry leaves improved skin hydration parameter slightly more than placebo cream in a month-long clinical trial on 25 volunteers (Tadić et al., 2021). However, in another very recent study, the bilberry fruit extract was found to reduce the sun protection factor upon incorporation into the tested sunscreens. This is a very unexpected and paradoxical result which requires further scientific attention (Ruscinc et al., 2022).

### 4.3 Pharmacological Activities of *V. myrtillus* Leaves

#### 4.3.1 Antidiabetic Activity

Bilberry leaf teas and extracts are a popular folk remedy in the treatment of diabetes. In Russia, for example, it is said to be the most commonly used herbal drug against diabetes (Shikov et al., 2021).

A thorough review of the early history of the bilberry leaf for diabetes was written by Helmstädter and Schuster in 2010. To briefly summarize, there was no mention of antidiabetic activity in the period sources before the end of 19^th^ century. From that point onward, several case studies and anecdotal evidence had begun to show up, followed by the first larger-scale experiments. During the first animal experiments (usually on dogs with surgically removed pancreas to induce partial or complete diabetes), case studies and clinical experiments in the 19^th^ and early 20^th^ century researchers observed various degrees of urinary and blood sugar decrease after pre-prandial administration of bilberry leaf teas or extracts, but some observed no effects at all. Subsequently, Frederick Madison Allen from the United States prepared an unspecified extract of bilberry leaves named *Myrtillin*. This extract has never been analyzed for the content of specific compounds. The resulting antidiabetic activity was assigned to a hypothetical “glucokinine” entity. Allen’s clinical studies brought very inconsistent results, his search for the active principle was unsuccessful and he was not able to continue funding his research. After World War II the research faded out, despite a couple of further attempts by different research teams which also yielded conflicting results. “Myrtillin” and “glucokinines”, however, kept being incorrectly referenced in later literature and can be still found in current articles and books (Helmstädter and Schuster 2010).

Over the past years the allegedly miraculous antidiabetic effect postulated by bilberry leaves has been coming up again, but the results continue to be contradictory at best. When administered to streptozocin-induced diabetic rats, a bilberry leaf extract reduced the plasma glucose levels by 26% (Cignarella et al., 1996). In another study on diabetic GK rats the bilberry leaf decoction lowered the occasional glycaemia (Ferreira et al., 2010) and, in a similar study, blood glucose level and glycated hemoglobin returned to normal in 50% of the diabetic rats (Sidorova et al., 2017). A study on prediabetic and diabetic mice showed that the extract from bilberry leaves inhibits *α*-glucosidase and *α*-amylase enzyme activity and prevents postprandial hyperglycaemia by slowing down the rate of saccharide digestion (Takács et al., 2020). The α-glucosidase inhibitory activity of the applied bilberry leaf extract was found to be comparable to acarbose in an *in vitro* experiment (Bljajić et al., 2017). However, in an oral glucose tolerance test in healthy rats, an alcoholic extract of *V. myrtillus* leaves unexpectedly caused an increase of the serum glucose levels compared to controls (Neef et al., 1995). To the best of our knowledge, no clinical study has been performed on this topic in the last 20 years.

## 5 Discussion

Many herbal remedies have had their mechanism of action elucidated by modern scientific approaches and continue to be beneficial for the treatment of various disorders in synergy with standard medication regimes. The current standing of bilberry fruits and extracts, in accordance with European Union herbal monographs, is still on the level of traditional use and more clinical research is necessary towards well-established use.

A complete lack of new clinical research can be observed for the use of bilberry fruits on venous insufficiency and microcirculation since the assessment report by European Medicines Agency from 2015 (European Medicines Agency 2015a). Older studies, while reporting generally positive results, are usually lacking in methodology and statistical analysis (as noted by the EMA assessment report itself), therefore the recommendations towards the use of bilberries for this indication should be treated with caution.

The same can be said for the use of bilberry leaf extracts for glycemic control and T2DM. In this case, dated and contradictory results are amplified by the striking dissonance: bilberry leaf extract is generally labeled as a “traditional remedy for diabetes”, yet there is no mention of it in the period sources, herbals and encyclopedias up until the end of the 19^th^ century. The latest animal models suggest the possible benefit of bilberry leaf teas and extracts as an α-amylase and α-glucosidase inhibitor, but these are yet to be verified by clinical studies. Contradictory clinical results for this application exist for the use of bilberry fruits as well; the improvement in some measured clinical parameters might be attributed to the general antioxidant and anti-inflammatory effects of the bilberry phenolics (Kolehmainen et al., 2012) but in the majority of the performed clinical trials there seems to be no specific benefit against T2DM, despite the fact that *in vitro* antidiabetic activities have been described for several isolated bilberry phenolic compounds.

When it comes to the use of bilberry fruit extracts for night vision and other eye-related disorders, it needs to be stated that the origin of this indication might have been a purposefully created hoax or a superstition. The systematic review of clinical studies showed no or only marginal benefit of supplementing bilberry fruit extracts to increase night vision (Canter and Ernst 2004). Similarly, only small improvements can be seen for diabetic retinopathy, ocular fatigue and dry eye symptoms.

**FIGURE 5:**
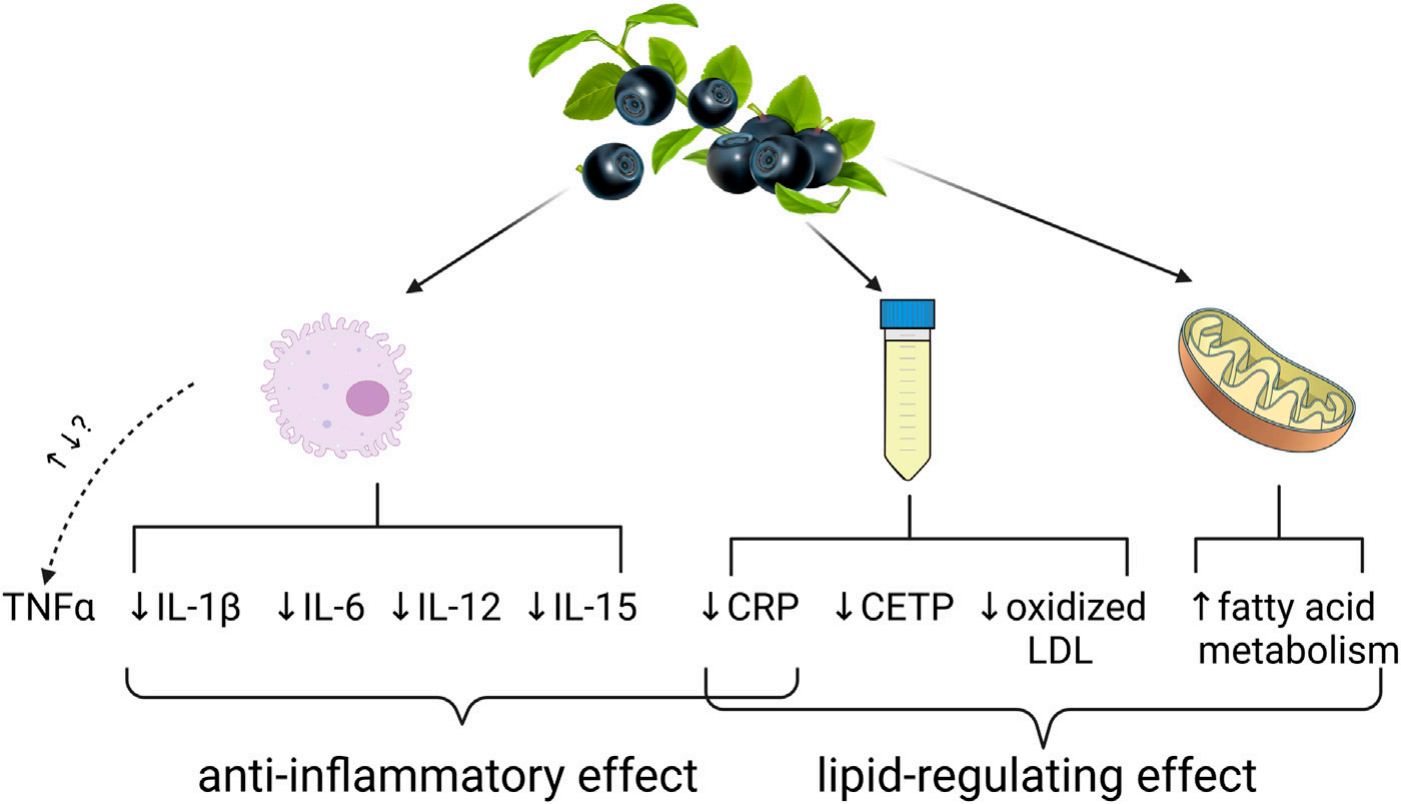
A summary of the theorized mechanisms of action of bilberry fruits and extracts in the treatment of inflammatory diseases and dyslipidemias.

Bilberry fruits and fruit extracts achieve the most positive clinical results in dyslipidemias and chronic inflammatory diseases such as oral mucosa inflammation, ulcerative colitis, metabolic syndrome and increased cardiovascular risk associated with elevated inflammatory serum values. So far, the consensus on the theorized mechanisms of action for the anti-inflammatory effect includes attenuation of pro-inflammatory cytokines IL-1β, IL-6, IL-12, IL-15 and C-reactive protein. The effects on TNF-α might pose a future challenge because both attenuation and induction was observed in cell cultures and clinical trials alike (Karlsen et al., 2010; Kolehmainen et al., 2012; Roth et al., 2014). The lipid-regulating effect is theorized to stem from the attenuation of the activity of plasma cholesteryl ester transfer protein (CETP) in plasma, upregulation of fatty acid metabolism in mitochondria and reduced oxidation of LDL. The reduction of CRP is also positively correlated with the reduction of plasma LDL (Qin et al., 2009; Zhu et al., 2013; Arevström et al., 2019) (Figure 5).

This makes bilberries a promising therapeutic for inflammatory disorders and dyslipidemia. However, further studies on the involved mechanisms, as well as larger randomized blinded trials are urgently needed.
