# Supplementary material for: Bilberries: Curative and Miraculous – A Review on Bioactive Constituents and Clinical Research
Source: Front Pharmacol. 2022 Jun 29;13:909914. doi: 10.3389/fphar.2022.909914 (PMC9277355; doi:10.3389/fphar.2022.909914)
Supplement: Supplementary file 1 [file Table1.pdf]

**Study / Condition**	**Design**	**Study group**	**Intervention**	**Outcomes**	**Statistically significant?**	**Comments**
Widén et al. 2015<br><br>Gingivitis	Randomized, placebo controlled trial	n = 24<br><br>Patients with gingivitis	250 or 500 g bilberries or placebo or standard therapy	Reduced inflammatory cytokine levels and bleeding on probing	Yes (for 500 g group)	-
Karlsen et al. 2010 Cardiovascular risk	Randomized, placebo controlled trial	n = 62<br><br>Subjects at elevated risk of cardiovascular disease	330 ml of bilberry juice or water<br><br>Daily for 4 weeks	Reduced C-reactive protein, IL-6, IL-15, and monokine induced by INF-γ<br>Increase of TNF-α<br><br>No change in oxidative stress markers or antioxidant status	Yes	-
Kolehmainen et al. 2012<br><br>Metabolic syndrome	Randomized controlled trial	n = 27<br><br>Overweight individuals with additional symptoms of metabolic syndrome	Equivalent of 400 g fresh bilberries (200 g of bilberry purée and 40 g of dried bilberries) or no intervention<br><br>Daily for 8 weeks	Reductions in several inflammatory parameters (C-reactive protein, IL-6, IL-12 and LPS)<br><br>Significantly lower inflammation scores<br><br>Glucose and lipid metabolism unchanged	Partially	-
Aboonabi et al. 2020<br><br>Metabolic syndrome	Open-label trial	n = 55<br><br>Healthy group and metabolic syndrome group	320 mg anthocyanin supplements (purified bilberry and blackcurrant anthocyanins)<br><br>Twice daily for 4 weeks	Reduced fasting serum glucose<br><br>Reduced serum triglycerides and LDL-C<br><br>Reduced high-sensitivity C-reactive protein<br><br>Decreased ADP-induced platelet activation configuration	Yes (in Metabolic syndrome group)	Lack of placebo control

Biedermann et al. 2012<br><br>Ulcerative colitis	Open-label pilot trial	n = 11<br><br>Patients with mild to moderate ulcerative colitis	Preparation made of dried bilberry powder and concentrated bilberry juice, 160 g (corresponding to 600 g fresh bilberries)<br><br>Daily for 6 weeks	63,4% patients achieving remission<br><br>Decrease in total Mayo score in all patients<br><br>Decreased fecal calciprotein levels	Yes	-
Lynn et al. 2018 Exercise-induced inflammation	Single blind, randomized, placebo-controlled trial	n = 19<br><br>Recreational runners	400 ml bilberry juice or control drink<br>Daily 5 days before, during and two days after a half marathon race	Mild increase of exercise-induced muscle soreness and of C-reactive protein levels in blood in bilberry group post-race	Yes	-
Hoggard er al. 2013<br><br><br>T2DM	Double-blinded, placebo-controlled cross-over trial	n = 8<br><br><br><br>Male subjects with T2DM controlled by diet and lifestyle alone or with impaired glucose tolerance	470 mg standardized bilberry fruit extract (Mirtoselect) or placebo<br><br>Single oral dose followed by a polysaccharide drink	Lower postprandial insulin and glucose blood levels compared to placebo<br><br><br>No change in gut, pancreatic or anti-inflammatory peptides	Yes	-

De Mello et al. 2011<br><br>T2DM	Randomized, controlled trial	n = 104<br><br>Individuals with impaired glucose metabolism and/or symptoms of metabolic syndrome	400g fresh bilberries as a part of diet high in fatty fish, bilberries and wholegrain products<br><br>Daily for 8 weeks	Lower fasting plasma glucose level<br><br>Insulin sensitivity remained unchanged	Yes	Bilberries were a part of a more complex dietary intervention
Chan et al. 2021<br><br>T2DM	Randomized, double-blind, placebo-controlled, cross-over intervention study	n = 20<br><br>Patients with T2DM controlled by oral medication	1400 mg of bilberry extract (25% anthocyanidins)<br>Once per day for 4 weeks	Reduced glycated hemoglobin<br><br>No change in other parameters	No	-
Canter and Ernst 2004<br>Night vision	Systematic review	All clinical studies before 2004	-	4 out of 5 randomized controlled trials found no significant effect<br>Other studies report improvements in some of the measured parameters	-	Trials without placebo control were
Scharrer and Ober 1981<br><br>Diabetic retinopathy	Open-label, placebo-controlled trial	n = 31<br><br>Patients with diabetic retinopathy	600 mg of unspecified bilberry fruit extract + 30 mg beta-carotene or placebo<br><br>Daily for 4 weeks	Reduced vascular permeability<br><br>Remission of changes of retinal vessels	n.a.	-

Perossini et al. 1987<br><br>Retinopathy (diabetic and hypertensive)	Randomized, double blind, placebo-controlled trial	n = 36<br><br>Patients with retinopathy	320 mg of a bilberry extract containing 25% anthocyanidins or placebo<br><br>Daily for 4 weeks	77 - 90% improvement (compared to placebo) in ophthalmoscopic and fluoro-angiographic anomalies	n.a.	No clear information about statistical analysis
Kim et al. 2008<br><br>Diabetic retinopathy	Prospective multicenter clinical trial	n = 88<br><br>Patients with diabetic retinopathy	Unspecified bilberry fruit extract 510 mg<br>Daily over one year	Gradual improvement in contrast sensitivity<br>Other parameters unchanged	Yes	Publication in Korean language
Steigerwalt et al. 2010<br><br>Elevated intraocular pressure	Open-label pilot trial	n = 79<br><br>Patients with elevated intra-ocular pressure	Dietary supplementation with Mirtogenol®: a combination of two phenolic extracts: 80 mg from bilberry (Mirtoselect®) (standardized to 36% anthocyanins) and 40 mg French maritime pine bark (Pycnogenol®) (standardized to 70% procyanidins), or Latanoprost eye drops, or combination<br><br>Once daily for 24 weeks	Intra-ocular pressure decrease took 4 weeks in latanoprost group and 24 weeks in Mirtogenol group, after which they reached the same levels<br><br>Comparable effects for gradually increasing central artery blood flow between groups	Yes	-

Anderson et al. 2011<br><br>Dry eye symptoms	Placebo-controlled trial	n = 22<br><br>Self-reported dry eye symptoms	160 mg of (unspecified) bilberry nutraceutical or placebo<br>Daily for 30 days	Ocular Surface Disease Index was improved compared to placebo<br><br>Tears break-up time and Schirmer's test not significant	Yes	Poster presentation with incomplete data
Kosehira et al. 2020<br><br>Ocular fatigue	Double-blind, randomized, placebo-controlled trial	n = 109	240 mg standardized bilberry extract (35% anthocyanins) or placebo<br><br>Daily for 12 weeks	Improvement in the tonic accommodation of the ciliary muscle during near-vision tasks on display terminal	Yes	-
Kamiya et al. 2013<br><br>Myopia	Prospective, randomized, placebo-controlled, cross-over trial	n = 30<br><br>With myopia, otherwise healthy	400 mg of yeast-fermented bilberry fruit extract or placebo<br><br>Daily for 4 weeks	Improvement in accommodation and mesopic contrast sensitivity<br><br>Other measured parameters unchanged	Yes	-
Arevström et al. 2019<br><br>Dyslipidemia and inflammation after myocardial infarct	Open-label randomized controlled trial	n = 50<br><br>Patients directly after myocardial infarct	Freeze-dried bilberry powder (40 g per day) taken with meals or no intervention<br>Daily for 8 weeks	hs-CRP unchanged between groups<br><br>Oxidized LDL improved in bilberry group<br><br>Improvement in a 6-minute walking test in bilberry group	Yes	-

Bryl-Górecka et al. 2020<br><br>Dyslipidemia and inflammation after myocardial infarct	Open-label randomized controlled trial	n = 50<br><br>Patients directly after myocardial infarct	Freeze-dried bilberry powder (40 g per day) taken with meals or no intervention<br><br>Daily for 8 weeks	Improvement in the blood profile<br><br>Reduced endothelial vesiculation	Yes	-
Qin et al., 2009<br><br>Dyslipidemia	Double-blind, randomized, placebo-controlled trial	n = 120<br><br>Patients with dyslipidemia	320 mg anthocyanin supplements (purified bilberry and blackcurrant anthocyanins)<br>Daily for 12 weeks	Decreased LDL and increased HDL plasma concentrations<br><br>Increased cellular cholesterol efflux to serum<br>No change in fasting glucose levels	Yes	-
Zhu et al., 2013<br><br>Dyslipidemia	Randomized, placebo-controlled trial	n = 150<br><br>Patients with hypercholesterolemia	320 mg anthocyanin supplements (purified bilberry and blackcurrant anthocyanins)<br>Daily for 24 weeks	Decreased CRP and IL-1b in plasma<br><br>Decreased LDL and increased HDL plasma levels<br><br>Fasting glucose levels unchanged	Yes	-
Habanova et al. 2016<br><br>Dyslipidemia	Pre–post intervention study	n = 65<br><br>Generally healthy subjects	150 g of frozen bilberries<br>3 times a week for 6 weeks	Decrease in total cholesterol, LDL and triglycerides, and a favorable increase in HDL	Yes	Lack of control group

Thomasset et al. 2009<br><br>Colorectal cancer	Open-label pilot trial	n = 25<br><br>Colorectal cancer patients scheduled for surgery	Standardized anthocyanin-rich bilberry fruit extract Mirtocyan (1400, 2800 or 5600 mg)<br>Daily for 7 days before surgery	Tumor cell proliferation decreased by 7%<br><br>Apoptotic index increased from 3.6% to 5.3%, regardless of the dosage	Yes	Lack of control group
Tadić et al. 2021<br><br>Skin hydration	Placebo-controlled trial	n = 25	Cream which incorporated both the oil from bilberry seeds and the extract from bilberry leaves or placebo cream<br>Daily for 30 days	Increase of the hydration of stratum corneum<br><br>Improved skin barrier function	Yes	-
